# Protective Immunity against Infection with Mycoplasma haemofelis

**DOI:** 10.1128/CVI.00581-14

**Published:** 2014-12-30

**Authors:** Chelsea A. E. Hicks, Barbara Willi, Barbara Riond, Marilisa Novacco, Marina L. Meli, Christopher R. Stokes, Christopher R. Helps, Regina Hofmann-Lehmann, Séverine Tasker

**Affiliations:** a School of Veterinary Sciences, University of Bristol, Bristol, United Kingdom; b Clinical Laboratory, Vetsuisse Facility, University of Zurich, Zurich, Switzerland; c Clinic for Small Animal Internal Medicine, Vetsuisse Facility, University of Zurich, Zurich, Switzerland; d Center for Clinical Studies, Vetsuisse Facility, University of Zurich, Zurich, Switzerland

## Abstract

Hemoplasmas are potentially zoonotic mycoplasmal pathogens, which are not consistently cleared by antibiotic therapy. Mycoplasma haemofelis is the most pathogenic feline hemoplasma species. The aim of this study was to determine how cats previously infected with M. haemofelis that had recovered reacted when rechallenged with M. haemofelis and to characterize the immune response following *de novo* M. haemofelis infection and rechallenge. Five specific-pathogen-free (SPF)-derived naive cats (group A) and five cats that had recovered from M. haemofelis infection (group B) were inoculated subcutaneously with M. haemofelis. Blood M. haemofelis loads were measured by quantitative PCR (qPCR), antibody response to heat shock protein 70 (DnaK) by enzyme-linked immunosorbent assay (ELISA), blood lymphocyte cell subtypes by flow cytometry, and cytokine mRNA levels by quantitative reverse transcriptase PCR. Group A cats all became infected with high bacterial loads and seroconverted, while group B cats were protected from reinfection, thus providing the unique opportunity to study the immunological parameters associated with this protective immune response against M. haemofelis. First, a strong humoral response to DnaK was only observed in group A, demonstrating that an antibody response to DnaK is not important for protective immunity. Second, proinflammatory cytokine interleukin-6 (IL-6) mRNA levels appeared to increase rapidly postinoculation in group B, indicating a possible role in protective immunity. Third, an increase in IL-12p35 and -p40 mRNA and decrease in the Th2/Th1 ratio observed in group A suggest that a Th1-type response is important in primary infection. This is the first study to demonstrate protective immunity against M. haemofelis reinfection, and it provides important information for potential future hemoplasma vaccine design.

## INTRODUCTION

The hemotropic mycoplasmas (hemoplasmas) are a group of mycoplasmal pathogens that adhere to the surface of red blood cells (RBCs) and are capable of inducing severe anemia (1). Three species of hemoplasma are recognized to infect both wild and domestic cats, with the occasional report of a fourth species (2). Mycoplasma haemofelis is recognized to be the most pathogenic species. Acute infection with M. haemofelis can result in severe hemolytic anemia, demonstrated in some experimental and natural infections by packed cell volumes (PCVs) falling below 15% (3, 4), leading to the onset of clinical signs, including pallor, lethargy, depression, pyrexia, anorexia, splenomegaly, and lymphadenopathy (3, 5, 6); without correct treatment, infection may result in death (7).

While antibiotics often help alleviate clinical signs, they are not always successful in clearing the infection (8–10). This can result in cats developing into chronic carriers, which remain persistently PCR positive at a low level in the absence of clinical signs of infection (11). Reactivation of infection remains a possible threat in chronic carriers (6), and repeated parasitemia in immunosuppressed animals has been reported (11). Recently a number of papers have documented the presence of hemoplasma infections, including the finding of a short sequence of DNA with 99% identity to a feline hemoplasma, in humans (12–16). Those in close contact with domesticated animals, such as veterinarians and farmers, and those in poor sanitary conditions are reported to be at an increased risk of hemoplasma infection (14). The failure of antibiotics to consistently clear these potentially zoonotic pathogens highlights the need to investigate the development of any protective immunity against these bacterial infections. The concept of protective immunity to M. haemofelis is one that has not been explored.

The aim of this study was to determine whether cats that have recovered from M. haemofelis infection are protected from reinfection with M. haemofelis and to describe the immunological changes during *de novo* infection and following rechallenge. All cats in this study were inoculated using the low-dose subcutaneous model developed by Baumann et al. (17), since it is considered to best mirror the proposed route of “natural infection” by arthropods or aggressive contact between cats (17).

## MATERIALS AND METHODS

### Animals, experimental design, and hematocrit.

Ten specific-pathogen-free (SPF)-derived male neutered domestic shorthaired (DSH) cats were used in this study. Group A comprised 5 M. haemofelis-naive cats (HBU2, HBV1, HCC2, HCD2, and HBZ2 [all 7 months old]), and group B comprised 5 cats (AKL4 and ZKA2 [7 years old] and KCU1, KCY2, and JCT2 [4 years old]) that had been previously experimentally infected with M. haemofelis (17) and allowed to recover. Four of the 5 group B cats (AKL4, ZKA2, KCU1, and JCT2) had required 10 mg/kg body weight/day oral doxycycline for up to 64 days, and 2 cats (AKL4 and KCU1) required 2 mg/kg body weight/day oral marbofloxacin for 13 days in conjunction with doxycycline, to become M. haemofelis negative by quantitative PCR (qPCR). Cat KCY2 became M. haemofelis qPCR negative naturally, without antibiotic treatment. All cats in group B were M. haemofelis qPCR negative on weekly sampling for a minimum of 7 weeks after antibiotic treatment had stopped before the start of the study. Groups A and B were housed separately throughout the study.

All 10 cats were inoculated on day 0 subcutaneously in the neck with 100 μl of dimethyl sulfoxide (DSMO) (20% [vol/vol]) preserved heparinized blood, containing approximately 10^3^ copies of M. haemofelis, diluted in sterile phosphate-buffered saline (PBS), as previously described (17). Rechallenge of the group B cats occurred 671 days after the initial inoculation of M. haemofelis.

Blood was collected into EDTA from all cats preinoculation on days −14, −7, and 0 and then postinoculation (p.i.) on days 1, 2, and 3, followed by once-weekly blood collections (on days 10, 17, 24, 31, 38, 45, 49, 56, 63, 70, 77, and 84 p.i.) up to day 84 p.i. for M. haemofelis qPCR, serology, cytokine analysis, hematocrit (HCT), and flow cytometry. Additionally blood was collected midweek (on days 6, 13, 21, 28, 35, and 42 p.i.) for hematocrit, M. haemofelis qPCR, and serology only.

Hemograms were run on a Sysmex XT-2000iv (Sysmex Corporation, Kobe, Japan) validated for cat blood samples (18). The reference range for hematocrit (HCT) was previously determined to be 33 to 45% (19). Cats were considered anemic if HCT was below 33%.

This study was approved by both the University of Bristol's Ethical Review Group (UB/12/021) and the Veterinary Office of the Canton Zurich, Switzerland (TVB 159/2010). The cats were kept in groups under ethologically and hygienically ideal conditions, as described previously (20).

### TNA extraction.

Total nucleic acid (TNA) was extracted using the MagNa Pure LC total nucleic acid isolation kit (Roche Diagnostics, Rotkreuz, Switzerland) from 100 μl of EDTA blood diluted in 100 μl of PBS. An extraction control of 200 μl of PBS was included with every extraction run to detect any cross contamination.

### Feline albumin gene internal control qPCR.

All samples were subjected to a feline albumin qPCR assay to confirm the presence of DNA within each sample (21). Those that contained less than 10^3^ copies of feline albumin underwent repeat extraction of a second sample and were again subjected to feline albumin qPCR. If repeat analysis failed to demonstrate adequate copies of feline albumin, the sample was not subjected to M. haemofelis qPCR.

### Mycoplasma haemofelis qPCR.

Total nucleic acid from each sample was screened for the presence and quantity of M. haemofelis DNA via an M. haemofelis-specific TaqMan qPCR, as described previously (5). In order to scale up the reaction for the number of copies of M. haemofelis present per ml of blood, the results of the qPCR were multiplied by 200, taking into account the volume of blood extracted, the volume of elution buffer that the DNA was eluted into, and the volume of DNA added to the PCR assay mixture, assuming 100% efficiency.

### Flow cytometry.

B220^+^ cells, identified B cells, and CD4^+^ and CD8^+^ cells were analyzed to identify the helper and cytotoxic T cells, respectively. CD4^+^ CD25^+^ and CD5^+^ major histocompatibility complex class II (MHC-II^+^) cells were used for markers of activated T cells. These staining combinations have all been used previously for feline research (19).

Fifty microliters of EDTA-treated blood was incubated with 5 μl of 1 of 3 different staining combinations of primary antibody: (i) fluorescein isothiocyanate (FITC)-conjugated mouse anti-feline CD25 antibody (kindly provided by G. Dean, Colorado State University) and R-phycoerythrin (RPE) mouse anti-feline CD4 (AbDserotec, MorphoSysAbD GmbH, Germany), (ii) FITC-conjugated mouse anti-feline CD5 (F43; Southern Biotech) and unconjugated IgG2b mouse anti-feline MHC-II (H34A; VMRD), or (iii) unconjugated IgG1 mouse anti-feline CD8 (FT2; Southern Biotech) and peridinin chlorophyll *a* protein (PerCP)-conjugated rat anti-mouse B220 (RA3-6B2; BD Bioscience).

Blood samples from each cat were used as isotype-matched control antibody and unstained negative controls. For those combinations of antibodies requiring secondary antibodies (combinations 2 and 3), only the primary antibody within the combination that required the addition of a secondary antibody (MHC-II [combination 2] and CD8 [combination 3]) was added in the first incubation step to prevent nonspecific binding. After incubation, 500 μl of VersaLyse (Beckman Coulter, Nyon, Switzerland) erythrocyte lysing solution was added to each sample tube, and the sample was vortexed and incubated in the dark at room temperature for 10 min. After complete erythrocyte lysis, each tube was centrifuged at 600 × *g* for 5 min to form a pellet of leukocytes, and the supernatant was discarded. The pellet was incubated with 50 μl of secondary antibody (RPE-labeled goat anti-mouse IgG2b [Southern Biotech, Birmingham, AL]) for the unlabeled anti-MHC-II antibody and allophycocyanin (APC)-labeled rat anti-mouse IgG1 (BD Pharmingen) for the unlabeled anti CD8 antibody or PBS containing 1% fetal calf serum (FCS) for 20 min in the dark. After incubation, the cells were washed in PBS containing 1% FCS and centrifuged at 1,200 × *g* for 5 min, and the supernatant was discarded. Those staining combinations that required a secondary antibody (combinations 2 and 3) then had 5 μl of their other primary antibody (CD5 [combination 2] and B220 [combination 3]) added at this point and were incubated in the dark for 30 min, followed by washing of the cells in PBS containing 1% FCS and centrifugation at 1,200 × *g* for 5 min. Leukocyte pellets were resuspended in 250 μl of PBS–1% FCS. Flow cytometry was performed on a Guava easyCyte HT sampling flow cytometer (Millipore Corporation, Billerica, MA) using the InCyte software. Gates were set using forward and reverse scatter for the lymphocyte population, and up to 10,000 events were acquired for each sample.

The absolute number of each lymphocyte cell subset was calculated as described previously (22), and to “correct” for differences in lymphocyte number between the two groups preinoculation, the results were expressed as the percentage change from preinoculation for each lymphocyte cell subset at each time point.

### Serology.

A recombinant M. haemofelis heat shock protein 70 (DnaK) enzyme-linked immunosorbent assay (ELISA) modified after that described by Barker et al. (23) was used, with ELISA plates (Thermo Scientific 96-well, vinyl, flat-bottom microtiter plates) coated with 100 μl of a 1:2,500 dilution of recombinant DnaK (rDnaK) synthesized by Barker et al. (23). Plasma samples were serially diluted 3-fold, from a 1:3 dilution to a 1:6,561 dilution, and each plate included a duplicated positive standard from Barker et al. (23). A 1:5,000 dilution of alkaline phosphatase-conjugated goat anti-cat IgG Fc (Jackson immunoresearch) secondary antibody (diluted 50% [vol/vol] in glycerol) was used in this study. Following the addition of the detection substrate, plates were incubated in the dark for 60 min, after which the optical densities at 405 and 492 nm (OD_405_ and OD_492_, respectively) were measured using a computer-assisted ELISA plate reader (Labsystems Multiskan EX Primary EIA v2.1-0 with Genesis v3.0; VWR International, Ltd., Lutterworth, United Kingdom).

Samples were analyzed by plotting the log of their optical density (OD) against the log concentration of the sample. The relative antibody level (RAL) was calculated by comparison with the standard dilution series, where the undiluted standard was given an RAL of 1,000. The mean of the results from each sample's dilutions was calculated to give the RAL. Cats were identified as having seroconverted when their RAL crossed the threshold of seropositivity, calculated as the mean of the preinoculation RALs for all cats in group A plus 3 standard deviations (SD) (23). Optical density readings of all dilutions at several time points from one cat in group B and some early time points of some individual cats in group A were just off the standard curve; these levels were seen to represent very low levels of antibody and for statistical analysis were represented by 0.

### mRNA extraction and cDNA synthesis.

One hundred microliters of EDTA-blood was mixed with 300 μl of mRNA isolation lysis buffer (mRNA isolation kit I; Roche Diagnostics) directly after blood collection and stored at −80°C until mRNA isolation was performed.

The isolation of mRNA was carried out using the MagNA Pure LC mRNA HS kit (Roche Diagnostics) according to the manufacturer's instructions. An extraction control of 100 μl of PBS was run alongside every batch extraction to detect any cross contamination. The mRNA was eluted in 25 μl of elution buffer and stored at −80°C until cDNA synthesis could be performed.

For each mRNA sample, two reverse transcription reactions were performed using the high-capacity cDNA reverse transcription kit from Applied Biosystems following the manufacturer's instructions and then the products were pooled and stored at −20°C until use.

### Cytokine mRNA analysis.

Relative levels of interleukin-4 (IL-4), IL-6, IL-10, IL-12p35, IL-12p40, gamma interferon (IFN-γ), and tumor necrosis factor alpha (TNF-α) mRNA were determined by qPCR. IL-12p35, IL-12p40, and IFN-γ were chosen as Th1 cytokines to determine if responses postinoculation were directed to a Th1 response. Conversely IL-4 and IL-10 were chosen as Th2 cytokines. IL-6 and TNF-α were analyzed to detect any proinflammatory response. IL-12p35 and IL-12p40 mRNA levels were determined separately as IL-12 is encoded by 2 separate genes, p35 and p40, which are situated on separate chromosomes and independently expressed (24).

The primers and probes had been designed previously (25), and these reactions were optimized for use on an Mx3005P qPCR system (Agilent, Berkshire, United Kingdom). Each PCR mixture consisted of 6.25 μl of GoTaq Hot Start polymerase (Promega, Southampton, United Kingdom), MgCl_2_ to a final concentration of 4.5 mM, forward and reverse primers at a concentration of 200 nM, probe at a concentration of 100 nM, 2.5 μl of DNA template, and water to a final volume of 12.5 μl. For each 96-well plate, 3 positive controls (of known threshold cycle [*C_T_*] values) and 3 negative water controls were also run.

The qPCRs were carried out on an MX3005P qPCR system. Incubation at 95°C for 2 min was followed by 45 cycles of 95°C for 15 s and then 60°C for 30 s for V-abl Abelson murine leukemia viral oncogene (ABL), zeta polypeptide (YWHAZ), IL-4, IL-12p35, IL-12p40, and TNF-α assays or 58°C for 30 s for IL-6, IL-10, and IFN-γ assays. Assays were run in duplicate for each cDNA sample.

The ABL and YWHAZ assays amplified the mRNA of two housekeeping genes, and all assays were normalized to these two housekeeping genes (19) using an adaption of the method used by Taglinger et al. (25). For each housekeeper (HK), a correction value was calculated using the formula HK correction value = a constant number − mean *C_T_* of the housekeeper. The correction value was then added to each result from the different cytokine assays: corrected *C_T_* = *C_T_* target + HK correction value. The relative copy number was determined by the calculation relative copy number = efficiency^Δ^^*CT*^, where Δ*C_T_* = 45 − corrected *C_T_*.

The mean of the 2 replicates of each cytokine mRNA was then calculated, followed by the averaging of the sets of normalized data from the 2 housekeepers for each cytokine.

To allow for differences in cytokine expression levels between the two groups at preinoculation, the percentage of change in cytokine mRNA expression levels postinoculation relative to preinoculation was calculated for each time point.

Following initial cytokine mRNA expression analysis, the ratios of IL-4 to IL-12p35 mRNA, IL-4 to IL-12p40 mRNA, and IL-4 to IFN-γ mRNA were calculated as indicators of Th2/Th1 ratios, as used previously (19).

### Statistical analysis.

The statistical software package IBM SPSS Statistics version 19 was used to perform statistical analysis. The data were not amenable to parametric testing, so we were unable to fit a model to the whole data. Mann-Whitney *U* tests (*P*_MWU_ values) were used to make comparisons between both groups at each time point for each dependent variable. Friedman's tests (*P*_Friedman_ values) were used to determine if there was a statistically significant change over time for each group, followed by the Wilcoxon signed rank test (*P*_Wilcoxon_ values) to determine where any change from preinoculation was significant. Significance was taken at *P* < 0.05. Difficulties exist in assessing the level of correction required for these multiple comparisons, as some correlation exists between the measurements at different time points. Thus, we have reported *P* values that are uncorrected for multiple comparisons and present these results as a general guide to identify where differences are likely to occur.

## RESULTS

### Rechallenged cats demonstrate protection against M. haemofelis infection.

**FIG 1:**
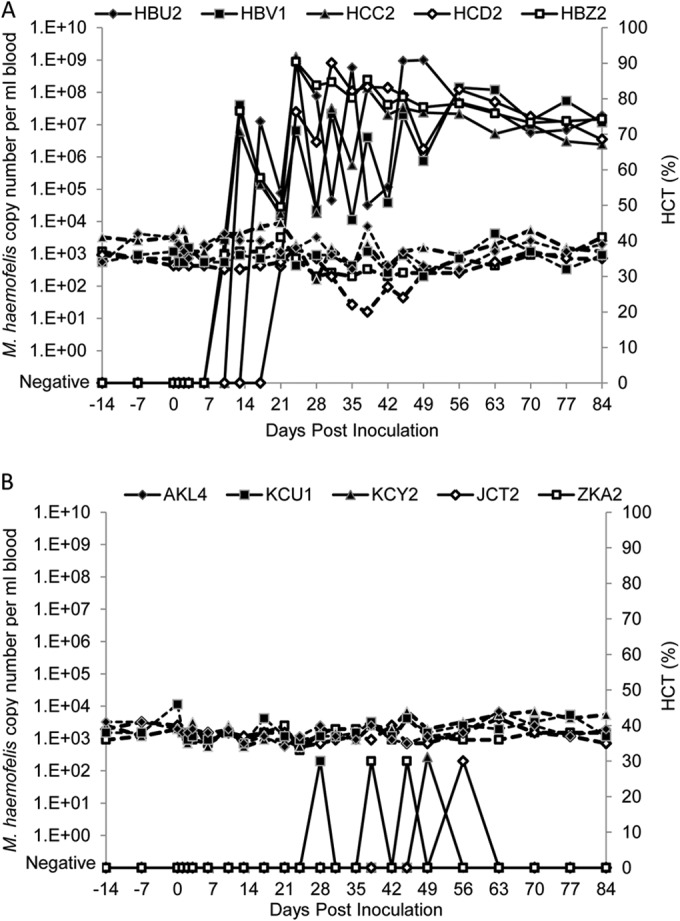
M. haemofelis copy numbers per ml of blood and hematocrit results during this study for groups A (A) and B (B). M. haemofelis copy numbers are reported as log values. All cats in group A became M. haemofelis PCR positive after inoculation by 21 days p.i., whereas the cats in group B remained PCR negative for the vast majority of the time, with only rare very low positive values recorded. Clear cycling was observed in the cats in group A: e.g., cat HCC2 showed a 4-log increase from day 21 to day 24 p.i., followed by a 4-log decline from day 24 to day 28 p.i. Dashed lines indicate hematocrit values.

All cats in group A (*de novo* infection) became M. haemofelis qPCR positive by day 21 p.i., with mean peak bacteremia reaching 5.79 × 10^8^ copies/ml blood at 24 days p.i. (Fig. 1A). A clear cycling of M. haemofelis copy numbers was observed (Fig. 1A).

In contrast, no evidence consistent with sustained multiplication of M. haemofelis was observed in group B (Fig. 1B). Three of the cats in group B (rechallenged with M. haemofelis: KCU1, KCY2, and JCT2) each gave just one positive M. haemofelis qPCR result, while cat ZKA2 (Fig. 1B) gave 2 positive results during the study; these low-level qPCR results showed M. haemofelis loads of only 265 copies/ml of blood or less (Fig. 1B). These low-level positive results were only just above the level of detection of the PCR assay, which is deemed to be 1 copy per PCR (thus equivalent to 200 copies per ml of blood). The results are suggestive of protection against M. haemofelis infection in the group B cats, as they indicate group B's ability to control the bacteremia, with no signs of bacterial levels rising further above the PCR detection limit. Each time point that gave a negative M. haemofelis qPCR result for group B was retested in duplicate to check for any low-level positive result; none were detected. The negative PCR status of the cats following identification of these very low levels is indicative of protection against M. haemofelis.

All cats in group A became anemic during the study (Fig. 1A), although this was mild. The lowest HCT recorded was 20% on day 38 p.i. (HCD2). The HCTs for group B all remained within the reference range (Fig. 1B).

### Naive infection is characterized by a reduction followed by a proliferation in lymphocyte cell subset numbers.

**FIG 2:**
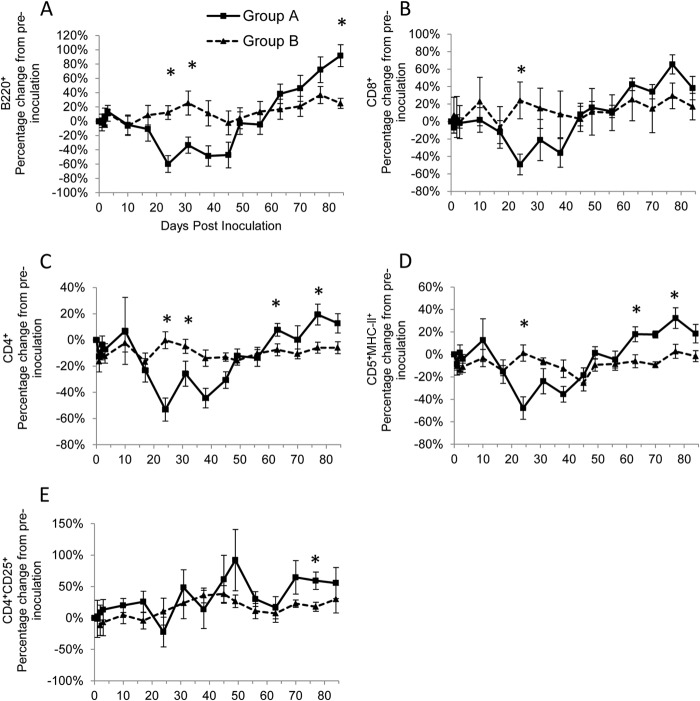
Percentage of change from preinoculation with B220^+^ (A), CD8^+^(B), CD4^+^ (C), CD5^+^ MHC-II^+^ (D), and CD4^+^ CD25^+^ (E) cells over the course of the experiment for groups A (■) and B (▲). Each value represents the mean result for the group, and error bars represent the standard error. Time point 0 represents the preinoculation value (mean from time points −14, −7, and 0). *, statistically significant difference between the two groups at *P*_MWU_ < 0.05.

In group A, all cell subsets showed a significant difference over time (*P*_Friedman_ ≤ 0.001) (Table 1), with a number of different cell subsets following a similar pattern: B220^+^, CD8^+^, CD4^+^, and CD5^+^ MHC-II^+^ (Fig. 2A to D). There was a significant decrease (*P*_Wilcoxon_ < 0.05) from preinoculation cell numbers at various time points early (up to around day 31 p.i.) in the study period (Table 1), with the nadir reached at day 24 p.i. (Fig. 2A to D); an increase in cell numbers then occurred (Fig. 2A to D), which corresponded with the time when antibody and cytokine levels increased dramatically in group A, such that significant increases (*P*_Wilcoxon_ < 0.05) from preinoculation numbers were seen at various time points toward the end of the study (Table 1). The numbers of CD4^+^ CD25^+^ cells in group A showed a similar but more varied cyclical pattern (Fig. 2E) and were significantly increased (*P*_Wilcoxon_ < 0.05) from preinoculation numbers on day 49 p.i. and toward the end of the study (Table 1).

**TABLE 1:** Statistical analysis of each test variable for the two groups in this study

Test variable	Cat group	Statistical analysis for any significant difference in variable over time (*P*_Friedman_ value)	Day(s) p.i. with significant differences (*P*_Wilcoxon_*) from preinoculation levels^*a*^
M. haemofelis rDnaK ELISA	A	χ^2^ = 82.859 (<0.001)	**24, 28, 31, 35, 38, 42, 45, 49, 56, 63, 70, 77, 84**
	B	NS^*b*^	Not performed
IL-12p35 mRNA	A	χ^2^ = 51.741 (<0.001)	**24, 31, 38, 45, 49**, **84**
	B	χ^2^ = 31.624 (0.007)	**17, 31, 45, 49**
IL-12p40 mRNA	A	χ^2^ = 35.082 (0.002)	**1, 3, 10, 24, 31, 38, 45**
	B	NS	Not performed
IFN-γ mRNA	A	NS	Not performed
	B	NS	Not performed
IL-4 mRNA	A	χ^2^ = 35.100 (0.002)	**2**, 63, 84
	B	χ^2^ = 26.382 (0.034)	**24, 31**, 77
IL-10 mRNA	A	χ^2^ = 52.094 (<0.001)	**17, 24, 31, 38, 45, 56, 63, 70**
	B	χ^2^ = 27.159 (0.027)	56
IL-6 mRNA	A	NS	Not performed
	B	NS	Not performed
TNF-α mRNA	A	χ^2^ = 28.200 (0.020)	
	B	χ^2^ = 29.382 (0.014)	**24, 49,** 84
IL-4/IL-12p35 mRNA	A	χ^2^ = 48.371 (<0.001)	31, 38, 45, 49, 63, 70, 84
	B	χ^2^ = 34.2 (0.003)	49, 56, 77
IL-4/IFN-γ mRNA	A	χ^2^ = 29.576 (0.014)	56, 63, 70
	B	χ^2^ = 25.129 (0.048)	63
B220^+^	A	χ^2^ = 54.706 (<0.001)	3, 24, 31, **63, 70, 77, 84**
	B	χ^2^ = 31.624 (0.007)	**77, 84**
CD8^+^	A	χ^2^ = 49.288 (<0.001)	24, **63, 70, 77**
	B	χ^2^ = 30.476 (0.010)	
CD4^+^	A	χ^2^ = 46.271 (<0.001)	24, 31, 38, 45
	B	NS	Not performed
CD4^+^ CD25^+^	A	χ^2^ = 36.918 (0.001)	**49, 70, 77, 84**
	B	χ^2^ = 28.588 (0.018)	**38, 45, 70, 84**
CD5^+^ MHC-II^+^	A	χ^2^ = 49.624 (<0.001)	24, 38, 45, **63, 70, 77**
	B	NS	Not performed

a Blood samples for cytokines and flow cytometry were collected on days 1, 2, 3, 10, 17, 24, 31, 38, 45, 49, 56, 63, 70, 77, and 84 p.i. Only those values that were significant at *P*_Friedman_ < 0.05 were subjected to the Wilcoxon signed-rank test. * indicates *z* = −2.023 and *P*_Wilcoxon_ = 0.043. Numbers in boldface indicate a significant increase in comparison to preinoculation levels, while numbers not in boldface indicate a significant decrease in comparison to preinoculation levels.

b NS, no significant difference.

Significant differences over time were identified in group B for the B220^+^, CD8^+^(*P*_Friedman_ ≤ 0.01), and CD4^+^ CD25^+^ (*P*_Friedman_ < 0.05) cell subsets, whereas CD4^+^ and CD5^+^ MHC-II^+^ cells did not differ over time (Table 1). In contrast with group A, the fluctuations in B220^+^, CD8^+^, and CD4^+^ CD25^+^ cell numbers were not marked in group B (Fig. 2A, B, and E), and fewer significant differences compared to preinoculation numbers were seen (Table 1).

Some significant differences (*P*_MWU_ < 0.05) in cell subsets were seen between groups A and B at various time points p.i. (Fig. 2); generally group A had lower levels of most of the cell subsets around day 24 p.i. but then had higher levels later in the study, around days 77 to 84 p.i.

### Protective immunity is not associated with a secondary antibody response to rDnaK.

**FIG 3:**
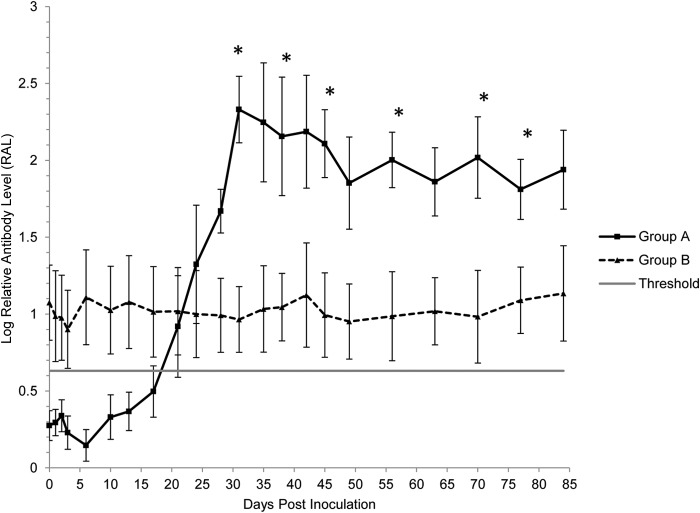
The humoral response to an rDnaK protein. Each value represents the mean log-transformed relative antibody level (RAL) for the group of cats, and error bars indicate the standard error. Time point 0 represents the preinfection value (mean RALs from time points −14, −7, and 0). *, statistically significant difference between the two groups at *P*_MWU_ < 0.05. The threshold represents seropositive status in group A *de novo*-infected cats.

Cats in group A seroconverted, on average, 21 days p.i. (Fig. 3). Relative antibody levels were increased significantly (*P*_Wilcoxon_ < 0.05) on day 24 p.i. compared to preinoculation and remained significantly (*P*_Wilcoxon_ < 0.05) higher throughout the remainder of the study (Table 1), with a maximal mean RAL reached on day 31 p.i. (Fig. 3). No significant changes in RAL in group B occurred over time (Table 1 and Fig. 3).

From day 31 p.i., levels of rDnaK antibodies were significantly (*P*_MWU_ < 0.05) greater in group A than those of group B and remained so for most of the remaining time points of the study (Fig. 3).

### Increased levels of both IL-12 mRNA subunits indicate a Th1-type response following naive infection.

**FIG 4:**
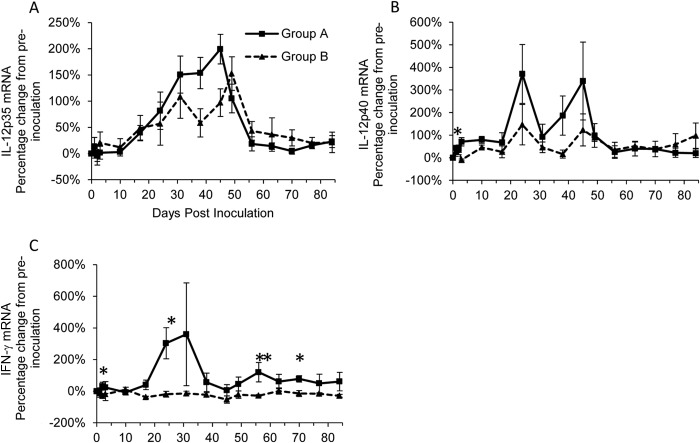
Percentage of change from preinoculation of Th1-type cytokines IL-12-p35 (A), IL-12p40 (B), and IFN-γ (C) over the course of the experiment for groups A (■) and B (▲). Each value represents the mean result for the group, and error bars represent the standard error. Time point 0 represents the preinoculation value (mean from time points −14, −7, and 0). *, statistically significant difference between the two groups at *P*_MWU_ < 0.05; **, significance at *P*_MWU_ < 0.01.

Levels of IL-12p35 mRNA in group A varied significantly (*P*_Friedman_ < 0.001) over time (Table 1), with levels being significantly (*P*_Wilcoxon_ < 0.05) higher than those at preinoculation midway through the study between days 24 and 49 p.i. (Table 1), reaching a peak on day 45 p.i. (Fig. 4A). Changes in IL-12p35 mRNA levels in group B largely mirrored those of group A (Fig. 4A), although levels were significantly higher (*P*_Wilcoxon_ < 0.05) than those at preinoculation at fewer time points (Table 1). No significant differences in IL-12p35 mRNA levels were found between groups A and B (Fig. 4A).

Levels of IL-12p40 mRNA in group A varied significantly (*P*_Friedman_ < 0.01) over time (Table 1), with levels being significantly (*P*_Wilcoxon_ < 0.05) higher than preinoculation levels both early (days 1, 3, and 10 p.i.) and midway (days 24 to 45 p.i., when a double peak occurred) (Fig. 4B) through the study period. In contrast there were no significant differences in IL-12p40 mRNA levels over time for group B (Table 1). IL-12p40 mRNA levels were significantly (*P*_MWU_ < 0.05) greater in group A than those in group B on day 3 p.i. only (Fig. 4B). No significant differences in IFN-γ mRNA levels over time were detected for either group A or B (Table 1). However, group A had significantly (*P*_MWU_ < 0.05) higher IFN-γ mRNA levels than group B on days 2, 24, 56 (*P*_MWU_ < 0.01), and 70 p.i. (Fig. 4C).

Significant increases from preinoculation in both subunits of IL-12 mRNA for group A suggest that a Th1-type response occurs following naive infection.

### IL-10 mRNA expression appears to show a Th2-type response in naive infection but not protective immunity.

**FIG 5:**
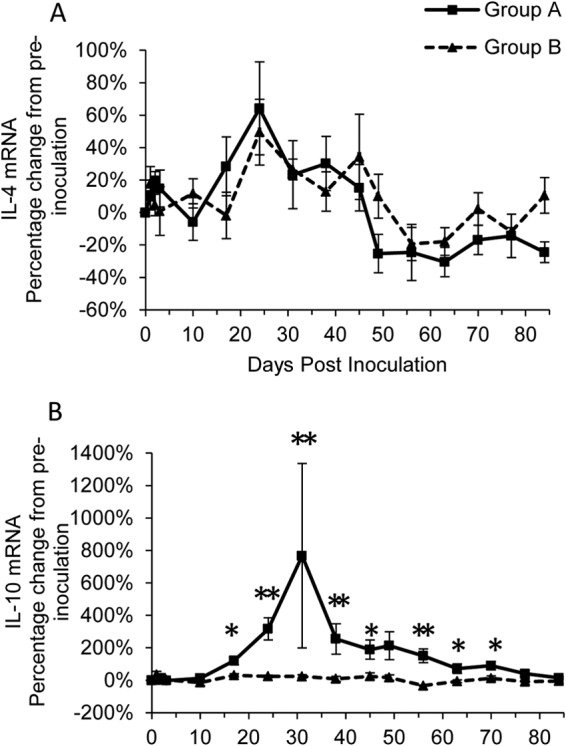
Percentage of change from preinoculation of Th2-type cytokines IL-4 (A) and IL-10 (B) over the course of the experiment for groups A (■) and B (▲). Each value represents the mean result for the group, and error bars represent the standard error. Time point 0 represents the preinoculation value (mean from time points −14, −7, and 0). *, statistically significant difference between the two groups at *P*_MWU_ < 0.05; **, significance at *P_MWU_* < 0.01.

IL-4 mRNA levels in group A were transiently significantly increased on day 2 p.i. (*P*_Wilcoxon_ < 0.05) (Table 1) but quickly fell back to preinfection levels before increasing, nonsignificantly, up to 24 days p.i. (Fig. 5A). The levels subsequently fell and were significantly (*P*_Wilcoxon_ < 0.05) decreased in comparison to preinoculation levels at the end of the study (Table 1 and Fig. 5A). The general pattern of IL-4 mRNA expression was similar in group B, although increases from preinoculation levels were significant at days 24 and 31 p.i. (*P*_Wilcoxon_ < 0.05) (Table 1). No significant differences were detected at any time point between groups A and B.

There was a significant (*P*_Friedman_ < 0.001) change in the level of IL-10 mRNA over time for group A, with levels significantly (*P*_Wilcoxon_ < 0.05) greater than those preinoculation on most p.i. sampling days between days 17 and 70 (Table 1), reaching a peak on day 31 p.i. (Fig. 5B). Group B also showed significant (*P*_Friedman_ < 0.05) differences in IL-10 mRNA levels over time (Table 1), but only levels at day 56 p.i. were significantly (*P*_Wilcoxon_ < 0.05) different and were decreased compared to preinoculation levels (Table 1). Levels of IL-10 mRNA were significantly (*P*_MWU_ < 0.05) increased in group A compared to group B from day 17 until day 70 p.i. (Fig. 5B). These results indicate a possible role of a Th2-type response in group A cats, in accordance with the production of antibodies against rDnaK.

### Potential role of proinflammatory cytokines in protective immunity.

**FIG 6:**
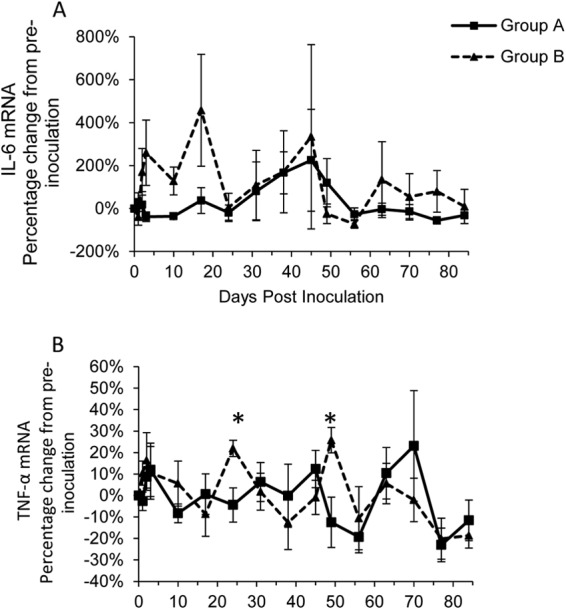
Percentage of change from preinoculation of proinflammatory cytokines IL-6 (A), and TNF-α (B) over the course of the experiment for groups A (■) and B (▲). Each value represents the mean result for the group, and error bars represent the standard error. Time point 0 represents the preinoculation value (mean from time points −14, −7, and 0). *, statistically significant difference between the two groups at *P*_MWU_ < 0.05.

There was a high level of variation of IL-6 mRNA between cats, and no statistically significant differences in levels over time were found either within (Table 1) or between (Fig. 6A) groups A and B. However, group IL-6 mRNA expression levels were (nonsignificantly) increased above preinoculation levels on 4 consecutive sampling days up to 17 days p.i. in group B, a sequence that was not observed in group A (Fig. 6A), suggesting a possible role of this cytokine in protective immunity.

Levels of TNF-α mRNA showed a significant (*P*_Friedman_ < 0.05) difference over time in group A (Table 1), but with no clear pattern for changes in expression (Fig. 6B) seen, and at no sampling point p.i. were levels significantly different from preinfection levels (Table 1). Group B also showed a significant (*P*_Friedman_ < 0.05) difference over time in TNF-α mRNA levels (Table 1), with significant (*P*_Wilcoxon_ < 0.05) peaks in expression on days 24 and 49 p.i. (Fig. 6B), corresponding to time points at which the levels of TNF-α mRNA in group B were significantly (*P*_MWU_ < 0.05) greater than those in group A (Fig. 6B). However, levels declined after each of these peaks, approximating preinfection levels.

### Decrease in Th2/Th1 ratios occurred in both naive infection and protective immunity.

**FIG 7:**
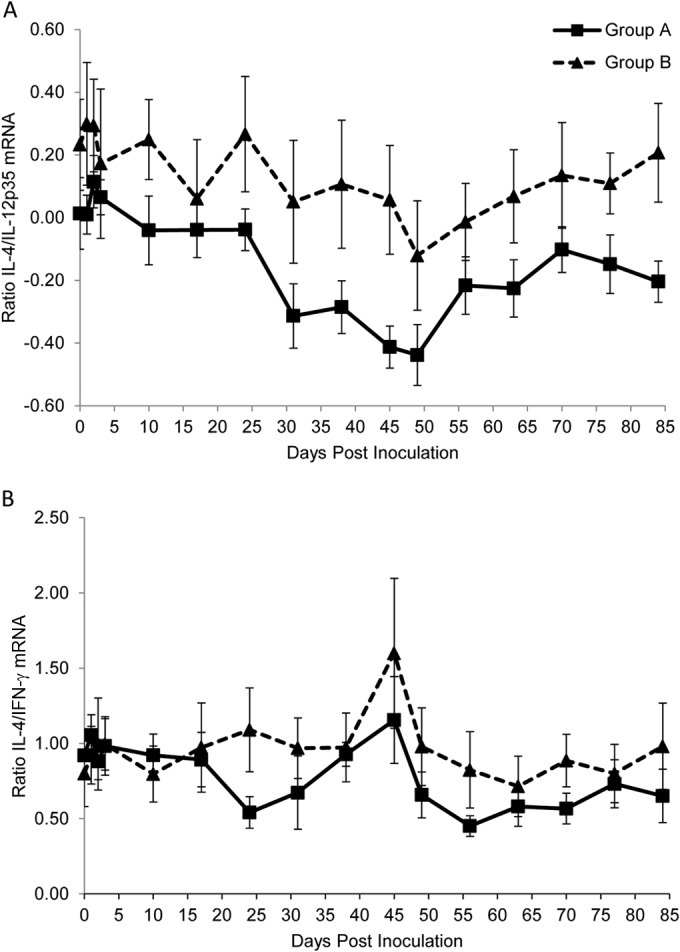
Ratios of IL-4 to IL-12p35 mRNA (A) and IL-4 to IFN-γ mRNA (B) in groups A (■) and B (▲) over the course of the study. Mean log-transformed values are plotted, and error bars represent the standard error.

The IL-4/IL-12p35 mRNA ratios showed a significant difference over time in both groups A (*P*_Friedman_ < 0.001) and B (*P*_Friedman_ < 0.01) (Table 1), with significant (*P*_Wilcoxon_ < 0.05) decreases compared to preinoculation levels at various time points between days 33 and 84 p.i. (Table 1). No significant differences between groups A and B were found at any time point (Fig. 7A). A similar pattern of expression was observed for the IL-4/IL-12p40 mRNA ratios (data not shown). The IL-4/IFN-γ mRNA ratios showed a significant (*P*_Friedman_ < 0.05) difference over time in both groups A and B (Table 1), with significant (*P*_Wilcoxon_ < 0.05) differences found compared to preinoculation levels toward the end of the study (Table 1), although no significant differences between groups A and B were found at any time point (Fig. 7B).

## DISCUSSION

While low, intermittent levels of bacteria, just above the detection limit of the PCR assay, were detected in 4 of 5 cats in group B, the absence of continued bacterial multiplication and the PCR-negative status thereafter are evidence of protection against M. haemofelis reinfection. These cats showed very occasional positive results by qPCR (in only 5 of the 105 p.i. samples from group B: once each for KCU1, KCY2, and JCT2 and twice for ZKA2). These low-level positive results were not thought indicative of sustained infection as there was no evidence of multiplication of M. haemofelis from levels just above the qPCR assay's limit of detection. These results represent levels that were very much lower (by ∼10^7^-fold) than those of the cats in group A, where the M. haemofelis loads showed continued sustained bacterial multiplication. Thus, we believe this study demonstrates, for the first time, protection from infection after an M. haemofelis rechallenge.

It is possible that the occasional positive qPCR results recorded in group B, just above the detection limit of the qPCR, represented laboratory contamination, but all qPCR-negative controls were appropriately negative in every PCR run, making this unlikely. Alternatively, these positive results could reflect M. haemofelis carrier cat status, but no positive qPCR results were seen in the time period (minimum 7 weeks) between cessation of antibiotic treatment and the start of the study (data not shown). We might also have expected to have seen many more than 5 M. haemofelis-positive PCR results in the group B cats were these true carrier cats. Additional qPCRs performed on each group B cat at its negative qPCR time points failed to yield any positive results, although it is possible that the identification of chronic carrier cats in group B may have been limited by the times when the cats were sampled. However, it is important to note that whether or not the cats in group B were chronically infected, they were still protected from succumbing to a rechallenge infection, as evidenced by the difference in results in group A compared to group B. We could have immunosuppressed the cats in group B before rechallenge to determine if carrier status existed by reactivation of the initial infection, as has been reported previously (11). However, time and ethical restraints meant that this was not possible during this study. An alternative explanation for the odd low-level PCR results could be that the bacteria used to inoculate the cats were being detected but were prevented from multiplying, although one might have expected the positive qPCR results to have been detected earlier than they were found. A control group consisting of 5 cats infected with hemoplasma-free blood in 20% DMSO would have been ideal to use as a comparison to the *de novo* and rechallenged cats. However, cost limitations precluded this in the present study, and previous studies have similarly not included such control cats (3, 17, 19).

In contrast, all cats in group A developed a high bacteremia and the characteristic cyclical nature of M. haemofelis bacterial loads, as previously described (8, 10, 17). There was a difference in age between the two groups of cats in this study, with group A comprising younger cats, and it is possible that this difference could have influenced the outcomes following inoculation with M. haemofelis. However, the older cats (group B) were first challenged with M. haemofelis at the ages of 3 and 6 years, and this had resulted in all cats becoming infected, with anemia developing in 4 out of the 5 cats (17); therefore, we would have expected similar results following rechallenge in this study. Previously, clinical signs of disease have usually developed at the time of the highest bacterial loads (3, 8, 11), but in the present study, all group A cats became only mildly anemic, showing drops in their hematocrit but no outward signs of clinical illness (data not shown), despite the known pathogenicity of this organism, illustrating that clinical signs do not necessarily indicate infection status. The young age of the cats in group A is unlikely to explain their lack of clinical signs as a previous study that involved experimentally infecting 7-month-old cats with M. haemofelis resulted in the development of severe anemia in 8 out of the 10 cats (3). The former studies (3, 8, 11) had used intravenous inoculation, unlike the low-dose subcutaneous inoculation used in the present study, although intravenous inoculation of M. haemofelis is not always associated with severe disease (8, 26). The subcutaneous method may more accurately mirror the natural route of infection but could be associated with lower pathogenicity. However, a previous low-dose subcutaneous M. haemofelis infection study reported 4 of 5 cats developing anemia, of which 3 showed signs of clinical illness (17). The same inoculum was used in this study as in the low-dose model study; this along with the high parasitemia observed in the group A cats should indicate that all 10 cats were effectively challenged. In this study, the aim was to determine whether cats were protected from infection rather than pathogenicity; therefore, we believe that the lack of clinical signs observed in the naive infection does not mean that the challenge was not effective for this study.

No known effective methods for preventing M. haemofelis infection exist, nor are data available regarding the immune responses that occur following infection, so the findings of the present study documenting prevention of reinfection and associated immunological changes represent an important advancement in our understanding of immunity to M. haemofelis infection. No studies have previously evaluated protection from rechallenge with this feline hemoplasma species, although a recent study did evaluate protection from rechallenge with the less pathogenic hemoplasma species “Candidatus Mycoplasma turicensis,” and apparent protection was reported (19). In pigs, a recombinant MSG1 (Mycoplasma suis GAPDH [glyceraldehyde-3-phosphate dehydrogenase]-like protein 1) vaccine failed to induce protective immunity against the porcine hemoplasma M. suis, despite inducing a strong immune response characterized by the induction of both IgG1 and -2 antibodies and the proliferation of splenocytes (27). Our study, documenting protection from M. haemofelis rechallenge, suggests that a vaccination approach involving attenuated whole organisms may offer a route to hemoplasma protection.

All cats in group A seroconverted to rDnaK by 28 days p.i., in agreement with previous studies (17, 23, 28). Interestingly, despite the strong humoral response produced during naive infection, the antibody levels to rDnaK did not rise and remained relatively constant throughout the study in group B cats. This suggests that the response to rDnaK is not important for protective immunity, but it may be that these slightly raised, albeit low, and constant levels of rDnaK antibody seen both preinoculation and p.i. provided long-lasting protection against M. haemofelis, or there was rapid clearance of any immune complexes generated. A similar lack of increase in rDnaK antibody levels was also seen in cats protected from “*Ca*. Mycoplasma turicensis” rechallenge following previous recovery from infection with this organism (19). It may be that other patterns of antibody production occur for other hemoplasma immunogenic determinants (29) that mediate protection, but testing methods to demonstrate such antibodies are not yet available.

Effector T cells expressing CD4 can be divided into type 1 and type 2 helper T cells (Th1 and Th2, respectively). The Th1 and Th2 paradigm is a useful model in which to determine the direction of the immune response following infections (30), with Th1 generally governing the clearance of intracellular pathogens, while Th2 controls extracellular pathogens (although cell subsets other than Th1 and Th2 are also involved). Evidence suggests that this model may also be of relevance in cats (31). The immunological changes in group A, as they became M. haemofelis qPCR positive, were overall suggestive of a Th1-type response. Even though IFN-γ levels did not change significantly (although they were significantly greater than those in group B at various time points), the increased mRNA levels of both IL-12p35 and IL-12p40 (and thus IL-12) p.i. and decreases in the IL-4/IL-12p35 and IL-4/IFN-γ mRNA ratios support the presence of a Th1 response in group A. Analysis of the IL-4/IL-12p35 and IL-4/IFN-γ mRNA ratios in group B cats also showed a decrease p.i.; however, in group B only IL-12p35 and not IL-12p40 mRNA levels increased significantly p.i. IL-12 is a heterodimer formed of two individually expressed subunits, p35 and p40 (32), and it is important that both subunits are analyzed together to determine the IL-12 bioactive expression, so we cannot conclude that IL-12 protein levels were increased in group B p.i. Additionally, group B had no changes p.i. in IFN-γ mRNA, making it unlikely that they showed a Th1 response.

It has been suggested that the Th2 response can be characterized through the production of antibodies and increases in IL-4 and IL-10 mRNA levels. IL-4 mRNA levels were raised in both groups A and B p.i., but no significant differences between the two groups occurred. IL-10 mRNA levels were raised p.i. in group A, and antibodies to rDnaK were also detected in this group, indicating some Th2 response activity following *de novo* infection. However, IL-10 can also be produced by regulatory T cells (Tregs); therefore, there is also potential of involvement of these cells in the group A response. IL-10 is an anti-inflammatory cytokine that is able to inhibit the action of Th1 cells as well as a number of other cell types involved in the immune response (33). The increased levels of IL-10 mRNA in group A throughout the study period suggest a role of this cytokine in limiting the inflammatory response to M. haemofelis and potentially preventing the clearance of the organism, as it has been shown to do for Leishmania major (34), leading to chronic M. haemofelis infection. The absence of antibody production and the presence of no increase in IL-10 mRNA levels in group B appear to show that a Th2-type response is absent following reinoculation. Surprisingly, these results were in contrast to those from the “*Ca*. Mycoplasma turicensis” rechallenge study, where the authors proposed that a Th2-type response p.i. in the protected cats was associated with the protection from reinfection (19). The reason for this contrast could be due to differences in the pathogenicity between the two species (5).

The proinflammatory cytokines TNF-α and IL-6 were the only cytokines in which mRNA levels appeared to increase from preinoculation in group B cats predominantly. TNF-α has been linked to the defense against some bacterial infections (35, 36). In this study, mRNA levels were raised significantly in group B at 2 separate time points. It might be expected that to confer protection from reinfection, the changes in the immune response must occur rapidly p.i. No such rapid significant changes were identified for any cytokine mRNA tested in the present study, although a nonsignificant increase in IL-6 mRNA expression was seen in group B. IL-6 is a pleiotropic cytokine that induces the generation of IL-17-producing Th17 cells, a third T helper cell division (37). It is possible that there is a lack of a skew toward either a Th1 or Th2 response in the group B cats, as IL-6 may be an indicator of a Th17 response, which provides defense against extracellular bacteria, as well as intracellular bacteria and fungi, via the activation of neutrophils (38). In order to define the role of Th17 cells in protective immunity to M. haemofelis, further studies evaluating the levels of IL-17 mRNA in rechallenged cats are required.

Our findings indicated that most lymphocyte cell subsets followed a similar pattern in group A, characterized by a decrease in the number of cells at the time of maximal bacteremia in comparison to preinoculation levels, and then an increase until the end of the study. The decrease is most likely explained by the cells migrating from the peripheral blood to the draining lymph nodes, where they become activated and proliferate, resulting in the increased levels of immune cells at the end of the study. Proliferation of the different cell subsets corresponded with the time of strong antibody production and increased cytokine response in the group A cats. The only subset in group A that failed to follow this path were the CD4^+^ CD25^+^ cells, which showed cyclical variability. CD25^+^ is a T cell activation marker. The results thus indicate a cyclic behavior of these activated T cells. When bacterial numbers are high, it is likely that the activated T cells are being sequestered to a site where they become further activated. In a previous “*Ca*. Mycoplasma turicensis” protection study, it was suggested that the dual expression of CD4^+^ and CD25^+^ might be used as a marker of regulatory T cells, which are able to suppress Th1 and Th2 responses, limiting the immune response to infection to prevent tissue damage and an excessive response (19, 39); however, CD4^+^ CD25^+^ expression alone is only an indirect marker of regulatory T cell populations (40); it is now known that Forkhead box P3 (FOXP3) should also be targeted to determine regulatory subsets (41), but such reagents were not available in this study. Although analysis of the CD4^+^ CD25^+^ data with the IL-10 mRNA levels suggests that there may be a role of CD4^+^ CD25^+^ FOXP3^−^ IL-10-producing regulatory T cells (42) in the group A cats; whether these cells exist within the cat is unknown. No changes in cell subsets for any of the markers chosen occurred in the first couple of weeks following inoculation in group B, making it unlikely that the changes observed conferred protection from infection.

In summary, once initial infection is apparently cleared, protective immunity develops against repeat M. haemofelis infection. Following naive infection, the immune response appears skewed toward a Th1 response. Although the mechanism of this protective immunity could not be defined in the present study, an early increase in proinflammatory cytokines does indicate their potential role. It is possible that other immunological parameters that constitute protection were missing from this experiment; however, this opens up the opportunity for further analysis of the cats protected from M. haemofelis by analysis of other parameters, such as T regulatory cells, Th17 responses, and other antigens. Despite a strong humoral response against rDnaK following naive infection, this does not appear to be important in protective immunity against M. haemofelis.
